# Modifiable lifestyle risk factors for stroke among a high risk hypertensive population in Greater Kampala, Uganda; a cross-sectional study

**DOI:** 10.1186/s13104-017-3009-7

**Published:** 2017-12-04

**Authors:** Mark Kaddumukasa, James Kayima, Jane Nakibuuka, Carol Blixen, Elisabeth Welter, Elly Katabira, Martha Sajatovic

**Affiliations:** 1 Department of Medicine, School of Medicine, Makerere University College of Health Sciences, P.O. Box 7072, Kampala, Uganda; 2 Neurological and Behavioral Outcomes Center, University Hospitals Cleveland Medical Center & Case Western Reserve University School of Medicine, 11100 Euclid Avenue, Cleveland, OH 44106 USA

**Keywords:** Stroke, Hypertension, Global health, Modifiable risk factors

## Abstract

**Objective:**

To describe the modifiable lifestyle risk factors for stroke among a high risk population for stroke. Africa suffers from rapid population growth, adoption of harmful western diets, and increased prevalence of hypertension and obesity.

**Results:**

A total of 440 study participants were screened and 87 individuals with hypertension plus at least one other known stroke risk factor were enrolled. The prevalence of hypertension and diabetes mellitus in the screened population was 19.7 and 1.8%, respectively. Among those with hypertension only 2.3% (2/87) had ever had serum lipid assessment. Seventy-two percent (68/87) had very high serum LDL-cholesterol, while 33.3% (29/87) had low levels of HDL-cholesterol, and 67.8% had mean blood pressures greater than 160/100 mmHg and 40% (35/87) were obese, with a BMI ≥30. Targeting individuals with modifiable stroke risk factors and implementing self-management programs may be a way to reduce stroke burden in Uganda.

## Introduction

Stroke is the second most common cause of mortality worldwide, and remains a leading cause of adult disability. Developing countries account for nearly 85% of global deaths from stroke [1–4]. In Africa, stroke accounts for 15% of hospital admissions and is a major contributor to mortality in both rural and urban areas [5]. Modifiable factors include hypertension, dyslipidemia, diabetes, cardiac disorders, sickle cell disease, high fat diet, sleep apnea, obesity, tobacco smoking, migraines, alcohol and drug abuse, oral contraceptive use, and lack of exercise [6–9]. The increasing prevalence of hypertension in low income countries represent a substantial public health problem with associated economic and social impacts [10]. Few studies have described the modifiable risk factors in Uganda [8, 11]. Information is lacking regarding modifiable risk factors among the stroke high risk populations in our settings. We describe the modifiable risk factors among a stroke high risk urban population in Mukono district, central Uganda.

## Main text

### Methods

This was part of a cross sectional study of 440 participants, assessing barriers and facilitators to stroke risk reduction and a cross-sectional population survey on stroke knowledge and attitudes in Greater Kampala, Uganda, [12, 13].

A total of 87 study participants were deemed to be high risk for stroke from the cross-sectional survey. For this study, a high risk for stroke was defined as being hypertensive with at least one other known modifiable stroke risk factor.

A modified expanded questionnaire based on the World Health Organization (WHO) STEPs questionnaire [14] was used in this study. Trained study nurses collected information including demographic data, family history of hypertension and diabetes mellitus, past and present medical history of stroke risk factors was collected and a blood sample for analysis. They assessed the lifestyle, physical activity, dietary history including salt intake, drug history and self-reported drug adherence. They also measured the height and weight using standardized protocols to determine the body mass index (BMI) [15]. Blood pressure was measured using standardized protocols between 8 am and 11 am, with an Omron automated sphygmomanometer model HEM-907 (Omron Healthcare Corp., Bannockburn, Illinois, USA), whose accuracy has been validated [16]. Hypertension was defined as a systolic blood pressure ≥ 140 mmHg and/or a diastolic blood pressure of ≥ 90 mmHg, or treatment with anti-hypertensive medication [17]. A point of care, capillary blood sample was obtained for measurement of the random blood sugar (RBS) level using a One Touch Ultra^®^ glucometer from Johnson and Johnson Company, United Kingdom.

Total cholesterol (TC), low-density lipoprotein cholesterol (LDL), and triglycerides (TG) were considered high if they were ≥ 200 mg/dL, ≥ 3.37 mmol/L, ≥ 110 mg/dL, respectively; while high-density lipoprotein cholesterol (HDL) was considered low ≤ 40 mg/dL. Obesity was defined as BMI ≥ 30 kg/m^2^, while BMI ≥ 25 kg/m^2^ was deemed overweight. Descriptive statistics of mean, frequency, and percentages were used to summarize data on socio-demographic variables and associated clinical factors. All statistical analysis was performed using STATA software version 12 (Stata Corporation, College Station, TX, USA).

### Results

Among the 440 participants screened and 19.7% (87/440) met the inclusion of having hypertension plus at least one other stroke risk factor. The majority (65.5%, 57/87) of the study participants were women and the median age (IQR) of the study participants was 54 (42–62) years. Table 1 illustrates the sample demographic and clinical characteristics among the 87 study participants with hypertension plus one or more risk factors for stroke. Nearly 66% of the enrolled sample reported hypertension as the most common known health condition in their family, followed by stroke (38%), diabetes mellitus (30%), and obesity (26%).

**Table 1:** Demographic and clinical characteristics in a community-recruited sample of 87 Ugandans with hypertension and at least 1 other stroke risk factor (N = 87)

Characteristic	n	% (n/N)
Gender		
Male	30	34.5
Female	57	65.5
Age in years, Median (IQR)	54 (42–62)	
Previous medical history		
Ever been diagnosed with diabetes	8	9.2
Ever been diagnosed with hypertension	59	67.8
Ever had blood test for lipid levels	2	2.3
Ever had heart attack	1	1.2
Ever diagnosed with heart disease or had heart surgery	0	0
HIV/AIDS infection	7	8.1
Lifestyle/social activities		
Currently using tobacco	5	5.8
Former smoker	7	8.1
Current alcohol consumption	26	29.9
Suspected alcohol problem	3	3.5
Alcohol abuse/dependency	0	0.0

Only 2.3% (2/87) had ever had laboratory assessment of serum lipids and 7 (8.1%) had concurrent HIV/AIDS co-infection.

Only 17.2% (N = 15) of individuals with hypertension had engaged in any sport in the previous 3 months before the study. Of these, only 13% (N = 11) engaged in vigorous physical exercise. The majority of individuals endorsed walking as their main form of physical activity.

Only 24.1% (21/87) of participants included fruits in their diets for 3 or more days in a typical week, while 29% did not include fruits in a typical week. Approximately one-third ate green leafy vegetables 3 or more days in a typical week, and 55.2% ate vegetables between 1 and 3 days in a typical week. The majority reported that vegetable oil was their most common type of oil/fat used in cooking. Ninety-three percent of the study participants (N = 81) reported that they usually added salt to their prepared food. Of these, half used the palm of their hand to measure added cooking salt, while 36% measured salt using their fingers (pinch of salt), and 6.9% used a tea spoon.

Eight-seven study subjects with hypertension were evaluated for stroke risk factors. The mean height (SD) was 159.8 ± 8.4 cm, with a range of 142–177 cm. The mean weight was 73, 14.5 kg, with a range of 45–110.2 kg. The mean pulse rate was 79.6, 13.6 beats per min (bpm), with a range of 42–129 bpm. The average mean blood pressure was 167.4/99.9 mmHg among these study participants. Seventy-two percent (68/87) had very high serum LDL-cholesterol and none had optimal levels. About 58% (50/87) had desirable levels less than 200 mg/dL of the total cholesterol, while 33.3% (29/87) had low levels of HDL-cholesterol. Only 3.5% (3/87) had high levels of fasting triglycerides (see Tables 2, 3). Blood pressure was well controlled in only 5 study participants (5.7%), while 67.8% had blood pressures more than 160/100 mmHg. The mean body mass index (kg/m^2^) was 28.7, 5.8 with a range of 16.2–45.1 kg/m^2^. Twenty-five percent (22/87) had a normal BMI, while forty percent (35/87) of the study participants were obese, thirty-one percent were overweight and one percent of the study participants were underweight.

**Table 2:** Physical and laboratory parameters among the 87 clinically evaluated patients with hypertension

	n (N = 87)	%
Blood pressure levels classifications (SBP/DBP) mmHg		
Normal: < 120/< 80	1	1.2
Prehypertension: 120–139/80–89	4	4.6
Hypertensive: ≥ 140/≥ 90	82	94.3
Hypertension, stage 1: 140–159/90–99	23	26.4
Hypertension, stage 2: > 160/> 100	59	67.8
Body mass index (BMI)		
Under weight	1	1.1
Normal weight; 20–24.9 kg/m^2^	23	26.5
Over weight; ≥ 25 kg/m^2^	28	32.2
Obese; ≥ 30 kg/m^2^	35	40.2
LDL-cholesterol		
Optimal: less than 2.59 mmol/L	0	0.0
Near/above optimal: (2.59–3.34 mmol/L)	3	3.5
Borderline high: (3.37–4.12 mmol/L)	1	1.2
High: (4.15–4.90 mmol/L)	4	4.6
Very high: greater than (4.90 mmol/L)	63	72.4
High LDL: ≥ 130 mg/dL (≥ 3.37 mmol/L)	68	78.2
Total cholesterol		
Desirable: less than 200 mg/dL (5.18 mmol/L)	50	57.5
Borderline high: 200–239 mg/dL (5.18 to 6.18 mmol/L)	18	20.7
High: 240 mg/dL (6.22 mmol/L) or higher	3	3.5
High TC: ≥ 200 mg/dL	21	24.1
HDL-cholesterol		
Low HDL: ≤ 40 mg/dL	10	11.5
Low level, increased risk: Less than 40 mg/dL (1.0 mmol/L) for men and less than 50 mg/dL (1.3 mmol/L) for women	29	33.3
Average level, average risk: 40–50 mg/dL (1.0–1.3 mmol/L) for men and between 50 and 59 mg/dl (1.3–1.5 mmol/L) for women	18	20.7
High level, less than average risk: 60 mg/dL (1.55 mmol/L) or higher for both men and women	33	37.9
Fasting triglycerides		
Desirable: less than 150 mg/dL (1.70 mmol/L)	58	66.7
Borderline high: 150–199 mg/dL (1.7–2.2 mmol/L)	10	11.5
High: 200–499 mg/dL (2.3–5.6 mmol/L)	3	3.5
Very high: greater than 500 mg/dL (5.6 mmol/L)	0	0.0
High TG: ≥ 110 mg/dL	26	29.9

**Table 3:** Laboratory tests and results of the 87 clinically evaluated patients with hypertension with one or more risk factor for stroke

Chemistry (mmol/L)	Mean (SD)	Min, max
Potassium	4.3 (0.4)	3.6, 5.7
Sodium	134.2 (8.9)	113, 148
Blood urea nitrogen (BUN)	4.2 (1.7)	1.7, 14.6

	Median (IQR)	Min, max
LDL-cholesterol (mmol/L)	109.7 (72, 133.4)	2.9, 220.3
HDL-cholesterol (mmol/L)	50.2 (42.4, 59.9)	1.1, 227.5
Triglycerides (mg/L)	104.9 (66.1, 134.1)	0.8, 315.4
Total cholesterol (mg/L)	111.4 (84.2, 207.8)	4.5, 830
Blood glucose (mg/dL)	95 (83, 106)	51, 262
Serum creatinine estimation (mmol/L)	91 (77, 102.3)	46, 510

Of the enrolled sample, only 55% (48/87) were on anti-hypertensive medications at the time of the survey. Nearly 22% (19/87) were taking the antihypertensive drug; Nifedipine, followed by Bendroflumethiazide, Atenolol and Captopril at 9.2, 6.9 and 3.5%, respectively. Only two study participants were receiving aspirin as an anti-platelet agent and only one study participant (1.2%) was receiving lipid lowering drugs. Twenty-six percent of the female study participants (15/57) were currently using hormonal contraception methods. Twelve percent (7/57) were using oral contraceptives while 14% (8/57) were using injectable contraceptives.

Among enrolled participants, only 12.6% self-reported that they never forget taking their medications while 10.3% (9/87) reported they always forget their medications. 11.5% reported they had missed their medication doses the day preceding the interview. Nearly 28% (24/87) had missed their doses in the last 2 weeks preceding the interview. Only 20.7% reported that they sometimes stop taking their medications because they feel their blood pressure is controlled.

### Discussion

Reducing stroke burden and helping people to make the behavioral changes needed to diminish their likelihood of stroke is urgently. Key findings of this study are that about 70% of the study participants, were aware that they had hypertension, but their blood pressure was elevated and were not involved in actively managing their hypertension. Poorly recognized and managed hypertension appears to be a key modifiable stroke risk factor while life-style issues such as improving diet can be an additional focus of effort. This cross-sectional study found that the majority (94.3%) of people with hypertension had blood pressures greater than 140/90 mmHg, among these 67.8% had JNC-VII, hypertension stage 2 levels despite being prescribed antihypertensive medications. Most individuals with hypertension had abnormal fasting blood cholesterol levels while only 3.5% had high serum triglycerides.

HTN remains one of the important modifiable risk factors for stroke [18–21]. Whereas antihypertensive agents are the most effective agent to reduce blood pressure, behavioral interventions such as improving knowledge and self-management approaches should be used synergistically with antihypertensive agents [22, 23]. The National Non-Communicable Diseases Risk Factor Survey reported a high prevalence of hypertension (28.5%) within central Uganda among adults and that only 7% were aware that they were hypertensive [24]. This study found a prevalence of 19.7% and of these 67.8% were aware that they were hypertensive. It is possible that the relatively high proportion of individuals who were aware of their hypertension reflects urbanization and education trends. Our study included only urban villages with no rural populations. A study in a similar area reported an age standardized prevalence of hypertension of 27.2% (95% CI 25.9–28.5), though two-thirds of the study population resided in rural settings. Earlier studies have shown that awareness and knowledge related to blood pressure are very poor in this area [24, 25]. Among the study participants, 67.8% had JNC-VII, hypertension stage 2 levels of > 160/100 mmHg despite the medications. This would mean either these study participants were not regularly taking their medications or the treatment being prescribed was ineffective in blood pressure control. Further studies are needed to explore this among this population.

The Adult Treatment Panel III (ATP III) guidelines recommend the initiation of non-pharmacological management of hypercholesterolemia with lifestyle modifications, such as balanced diet, physical activity, and smoking cessation, for patients with total cholesterol of above 200 mg/dL [26]. Only one study participant was taking lipid lowering medications yet over half had abnormal serum lipids. No studies have described the prevalence of dyslipidemia among hypertensives in Uganda, however some studies looking at stroke patients and diabetics have reported prevalence of 17.8 and 20.5% [27, 28]. Studies in similar settings have reported higher levels of dyslipidemia among hypertensives up to 60% in Nigeria, [29]. There is an urgent need for attending physicians to routinely test the serum lipid levels among their patients with hypertension.

Current guidelines recommend increasing physical activity as a means to prevent hypertension, [30–32] and regular exercise is associated with up to a 70% reduced risk of ischemic stroke later in life [33]. In contrast to individuals at risk for stroke in other settings, many Ugandans walk long distances as their primary mode of transport. This may be sufficient exercise for some individuals, although urbanization and greater reliance on automobile or motorcycle transport may heighten activity-related stroke risk in the future among Ugandans.

Diet that includes frequent consumption of high quality fats (polyunsaturated and monounsaturated fats primarily from plant sources and fish), fruits, vegetables, and whole grains are associated with large reductions in CVD risk [34–36]. Only 24.1% of the study participants included fruits in their diets for 3 or more days in a typical week, while 28% did not include any fruits in their diets. Only 30% ate green leafy vegetables 3 or more days in a typical week. It is not entirely clear if the relatively high consumption of starchy foods and low consumption of fruits and vegetables is due to lack of knowledge vs. lack of access to healthier foods that may be beyond the financial reach of many Ugandans.

In conclusion, addressing modifiable lifestyle risk factors should be routinely integrated into primary hypertension care especially in primary health care facilities to reduce the risk of stroke among high risk populations.

## Limitations

Our study is limited by the fact that it was cross-sectional, used close-ended questions, and was confined to a fairly limited geographic area. The sample size of 87 is a major limitation for this study and might not provide sufficient inferences to make robust conclusions. Because participants of this study were residents of urban areas, the findings of this paper are not likely to applicable to the whole nation or rural settings within Uganda. The data collected from survey respondents was not adjusted to represent the population from which the sample was drawn.
